# International Text-Book of Surgery

**Published:** 1902-10

**Authors:** 


INTERNATIONAL TEXT-BOOK OF SURGERY.—Second edition, thoroughly revised and enlarged. In two volumes. By American and British authors. Edited by J. Collins Warren, M. D., LL.D., F. R. C. S. (Hon.), Professor of Surgery, Harvard Medical School, and A. Pearce Gould, M. S., F. R. C. S., of London, England. Second edition, thoroughly revised and enlarged. Volume I. General and operative surgery. Royal octavo of 965 pages, with 461 illustrations, and 9 full-paged lithographic plates. Volume II. Special or regional surgery. Royal octavo of 1122 pages, with 499 illustrations, and 8 full-paged colored lithographic plates. Philadelphia and London: W. B. Saunders & Co. 1902. Cloth, $5.00, net; sheep or half Morocco, $6.00, net.

The editors of these excellent volumes have no reason to apologize for making an addition to the list of treatises on surgery. Their work is a masterly production, thoroughly up-to-date, and makes its appearance at an opportune time. With reference to the need existing at the present time for additional works of reference on the subject, and for revised editions of old ones, Drs. Warren and Gould say "Modern surgery is still in the transition stage of development. The art and science of surgery is advancing rapidly, and the number of workers is now so great and so widely spread through the whole of the civilized world that there is certainly room for another work of reference which shall be untrammeled by many of the traditions of the past, and shall, at the same time, present with due discrimination the results of modern progress."

Having this in mind, they have, indeed, given to the profession an up-to-date and thoroughly reliable treatise covering the entire field of modern surgery, among the contributors to which are many of the foremost surgeons of this country and England. Volume I, for example, contains contributions from the following: Richard C. Cabot, on the "Surgical Pathology of the Blood"; Charles McBurney, on "The Technique of Aseptic Surgery"; J. C. Da Costa, on "Minor Surgery"; DeForest Willard, on "Diseases of the Joints," and W. W. Cheyne, of London, on "Diseases of the Bones," and contributions from many other equally distinguished men.

Among the contributors to Volume II are A. P. Gould, John B. Deaver, Wm. T. Bull, the late Christian Fenger and others.

Every general practitioner, as well as every surgeon, will profit by the reading of Dr. McBurney's instructive chapter on "The Technique of Aseptic Surgery." It contains many valuable suggestions not to be found in other text-books. He begins by describing the best approved methods for preparing the operating room, for sterilizing dressings, and then considers the question of the sterilizing of the site of the wound, and the hands of the operator and his assistants, etc. In short, he covers every detail connected with the technic of surgical operations in general. In considering the methods of hand sterilization he says that no hand can be made absolutely sterile by any of the known methods, and ends by advising operators to use well fitting rubber gloves in every case, advice which he has himself followed since 1897.

By way of revision, a number of the chapters have been entirely rewritten in the light of all of the knowledge recently gained on the several subjects with which they treat.

The illustrations being numerous (977) and exceedingly well done, constitute a valuable feature.

Being printed in two volumes and carefully indexed, the 2087 pages of the work are made easy of reference. R.

